# The effects of landscape change on habitat quality in arid desert areas based on future scenarios: Tarim River Basin as a case study

**DOI:** 10.3389/fpls.2022.1031859

**Published:** 2022-10-25

**Authors:** Tianju Zhang, Yaning Chen

**Affiliations:** ^1^ State Key Laboratory of Desert and Oasis Ecology, Xinjiang Institute of Ecology and Geography, Chinese Academy of Sciences, Urumqi, China; ^2^ University of Chinese Academy of Sciences, Beijing, China

**Keywords:** FLUS model, InVEST model, partial least squares regression, anthropogenic factors, land use land cover, landscape dynamics

## Abstract

Human activities have caused spatiotemporal patterns of land use and land cover (LULC) change. The LULC change has directly affected habitat quality (HQ) and ecosystem functions. Assessing, simulating, and predicting spatiotemporal changes and future trends under different scenarios of LULC-influenced HQ is beneficial to land use planners and decision-makers, helping them to formulate plans in a sustainable and responsible way. This study assesses and simulates the HQ of the Tarim River Basin (TRB) using the future land use simulation model (FLUS), the Integrated Valuation of Ecosystem Services and Trade-offs (InVEST) model, and partial least squares regression (PLSR). Since 2000, the TRB has experienced a declining trend in HQ from 0.449 to 0.444, especially in the lower elevations (740-2000m) and on sloped land (<10°). The decline will continue unless effective and sustainable plans are implemented to halt it. Agricultural and settlement areas have a lower HQ and a higher degree of habitat degradation than native habitats. This shows that the expansion of oasis agriculture (with an annual growth rate of 372.17 km^2^) and settlements (with an annual growth rate of 23.50 km^2^) has caused a decline in native habitat and subsequent habitat fragmentation. In other words, changes in LULC have caused a decline in the HQ. Moreover, there is a significant negative correlation between HQ and urbanization rate (p<0.01), and the PLSR also indicate that number of patches (NP), area-weighted mean fractal dimension index (FRAC_AM), percentage of landscape (PLAND), and largest patch index (LPI) were also important contributors to worsening the HQ. Therefore, the TRB urgently needs appropriate strategies to preserve its natural habitats into the future, based on the ecological priority scenario (EPS) and harmonious development scenario (HDS), which can help to maintain a high-quality habitat.

## 1 Introduction

Biodiversity is closely related to the development of human society and provides a material basis for human survival (Hou et al., 2021; He et al., 2022). It is well known that biodiversity and ecosystem services are inextricably linked, and they affect each other (Mengist et al., 2021). -i.e. the degradation of ecosystem resulted in the loss of biodiversity, in turn, the loss of biodiversity would resulted in the degradation of ecosystem (Yohannes et al., 2021). Habitat quality (HQ), as a reliable proxy to assess biodiversity, refers to the ability of an ecosystem to provide goods and services (Gomes et al., 2021). However, HQ has been threatened by human activities (Yang, 2021), causing habitat fragmentation, loss of biodiversity, and degeneration of natural habitats (Tang et al., 2020). Since entering the Anthropocene (a proposed geological period), Earth has undergone further changes due to human activities (Daru et al., 2021) that have disrupted the ecological balance and caused loss of ecosystem services. Despite most countries and organizations have taken necessary measures to halt it (Li et al., 2018), however, this trend of ecological imbalance and decline in biodiversity has not been curbed, which is still a matter of urgency (Hou et al., 2021). Assessing HQ under various scenarios would help to develop appropriate strategies to protect ecosystems and biodiversity. To do this, modelling could be used to estimate spatiotemporal dynamics of HQ (Yohannes et al., 2021). Integrated Valuation of Ecosystem Services and Trade-offs (InVEST) is a geographic information system (GIS)-based model that has been proved to be an effective and mature model in assessing HQ (Agudelo et al., 2020). This model uses HQ as a proxy for biodiversity, which simulates the combined effects of LULC and human activities on ecosystems (Upadhaya et al., 2019). It can quantitatively evaluates ecosystem functions and ecosystem services and realize a spatial display (Sharp et al., 2018). Moreover, it can easily execute dynamic change analysis and assessments under different scenarios (Caro et al., 2020; Wang et al., 2022). Therefore, it has been widely used (Mengist et al., 2021; Yohannes et al., 2021; Upadhaya et al., 2019; Wang et al., 2022).

Landscape pattern, which is the spatial arrangement and combination of landscape elements (Ma et al., 2022; Zhong et al., 2022), reflects the spatial distribution and heterogeneity of landscape (Fu et al., 2022; Ma et al., 2022) that either directly or indirectly influences the ecosystem (Dadashpoor et al., 2019). Currently, many natural ecosystems have been replaced by human-dominated landscapes (Tscharntke et al., 2012). By investigating landscape patterning, a deeper understanding of its impact on HQ, biodiversity, and ecosystem can be obtained. In general, changes in landscape pattern are more likely observed through changes in land use and land cover (LULC), as LULC changes lead to landscape pattern changes (Dadashpoor and Alidadi, 2017; Dadashpoor et al., 2019). Therefore, analysing LULC changes contributes to a better overall understanding of changes in landscape patterns (Nagendra et al., 2004; Dadashpoor et al., 2019).

The rapid socioeconomic development, industrialization, agricultural expansion, and urbanization have exacerbated the pace and pattern of LULC changes around the world (Tang et al., 2020; Yang, 2021). LULC change is an important factor threatening HQ that can cause change in the landscape structure and HQ, modifies habitat composition and configuration, leading to an alteration in species diversity (Zhang et al., 2020). For example, the loss of species, the decrease in biodiversity, the fragmentation of habitat, and the decline in ecosystem services function (Mengist et al., 2021). Therefore, LULC changes represent a significant driver in HQ decline (Zhang et al., 2020). It is also worth noting that climate change increases the complexity of the impacts of LULC changes on HQ and ecosystems (Pereira, 2020; Gomes et al., 2021).

Assessing, simulating, and predicting changes in LULC is beneficial to land use planners and decision-makers, helping them to formulate plans in a sustainable and responsible way (Jayanthi et al., 2021). Models are especially useful for addressing this problem. Numerous models have been developed in recent years, the most popular of which are cellular automata (CA), artificial neural network (ANN), Markov, logistic-CA, and ANN-CA (Lin et al., 2020; Zhang et al., 2021). However, most of these models cannot meet the needs of multiple LULC simulations (Liu et al., 2017). Multi-scenario simulation can better reflect its real dynamic change process. We try to evaluate the habitat quality of the Tarim River Basin through multi-scenario simulation, and promote the ecological restoration and high-quality development of ecological environmental protection. Moreover, climate change and ecological degradation have long-term effects that alter the natural landscape dynamics (Liu et al., 2017), but those factors are not well addressed in the aforementioned models. The future land use simulation (FLUS) model was developed in response to these shortcomings in simulation models (Liu et al., 2017). It interactively combines ANN and a CA model for multiple land use dynamic simulations. It has been proven effective for projecting complex LULC changes under different scenarios and thus has been widely used (Liang et al., 2018).

The Tarim River Basin (TRB), located in the arid area of northwest China, is an important socio-economic and ecological fragile area, which plays a significant role in the strategy of economic development and biodiversity conservation (He et al., 2022). However, the TRB’s recent rapid growth in population and urbanization, along with the expansion of its oasis agriculture and residential area and the increase in desertification, have all greatly changed the region’s land use patterns, directly affecting both HQ and biodiversity. Therefore, assessing, simulating and predicting spatiotemporal changes and future trends under different scenarios of LULC-influenced HQ is beneficial to land use planners and decision-makers, helping them to formulate plans in a sustainable and responsible way. However, most of the research on HQ in the TRB focuses on assessing the historical evolution and current status, and there is inadequate research on future multi-scenario simulations and predictions.

The study uses InVEST HQ module and FLUS models to assess and predict HQ change, and partial least squares regression (PLSR) to investigate the impacts of LULC landscape change on the HQ of the TRB. The objectives of the research are to: (1) simulate the temporal and spatial changes of HQ in historical and future periods; (2) quantify the relationships between HQ and LULC/landscape changes; (3) identify areas with poor HQ and speculate on possible causes of HQ degradation.

## 2 Materials and methods

### 2.1 Study area

**Figure 1:**
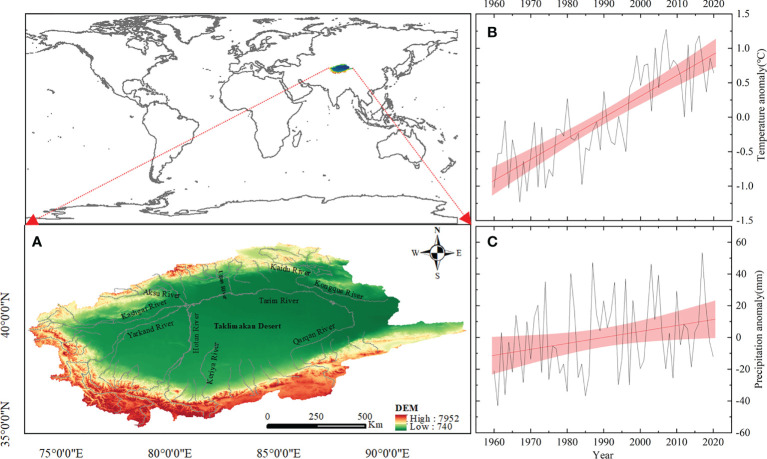
Map of the study area **(A)**, temperature trend **(B)** and precipication trend **(C)**.

The TRB is situated in a mid-latitude area that covers 73.40° to 93.65°E longitude and 34.80° to 43.35°N latitude (**Figure 1A**). The basin, which is flanked by Mountains (Kunlun and Tianshan), includes nine water systems and the Taklimakan Desert (the China’s largest desert) (Xue et al., 2019). It has a typical temperate continental climate (Wang et al., 2021). The mean annual precipitation and potential evaporation is about 50 mm and 2300-3000 mm, respectively (Liu and Yin, 2020). Compared to precipitation, the temperature has risen sharply in the past 20 years, which is in a highly fluctuation state (**Figures 1B, C**). The TRB encompasses 5 states and 42 counties, and oasis agriculture is the basis of livelihood.

The Tarim River is the China’s largest inland river (Wang et al., 2021). However, recent anthropogenic impacts and climate variability have caused some serious ecological and environmental issues, such as the drying of tributaries, dramatic declines in groundwater level, degradation of habitat and natural vegetation, desertification, and salinization of soil (Fu et al., 2021; Wang et al., 2021). In addition, plant species are relatively poor in this region. The dominant natural plants are typical to arid ecosystems and mainly include *Tamarix chinensis*, *Populus euphratica*, *Alhagi pseudalhagi* and *Phragmites communis* (Wang et al., 2021).

### 2.2 Data source and preprocessing

**Figure 2:**
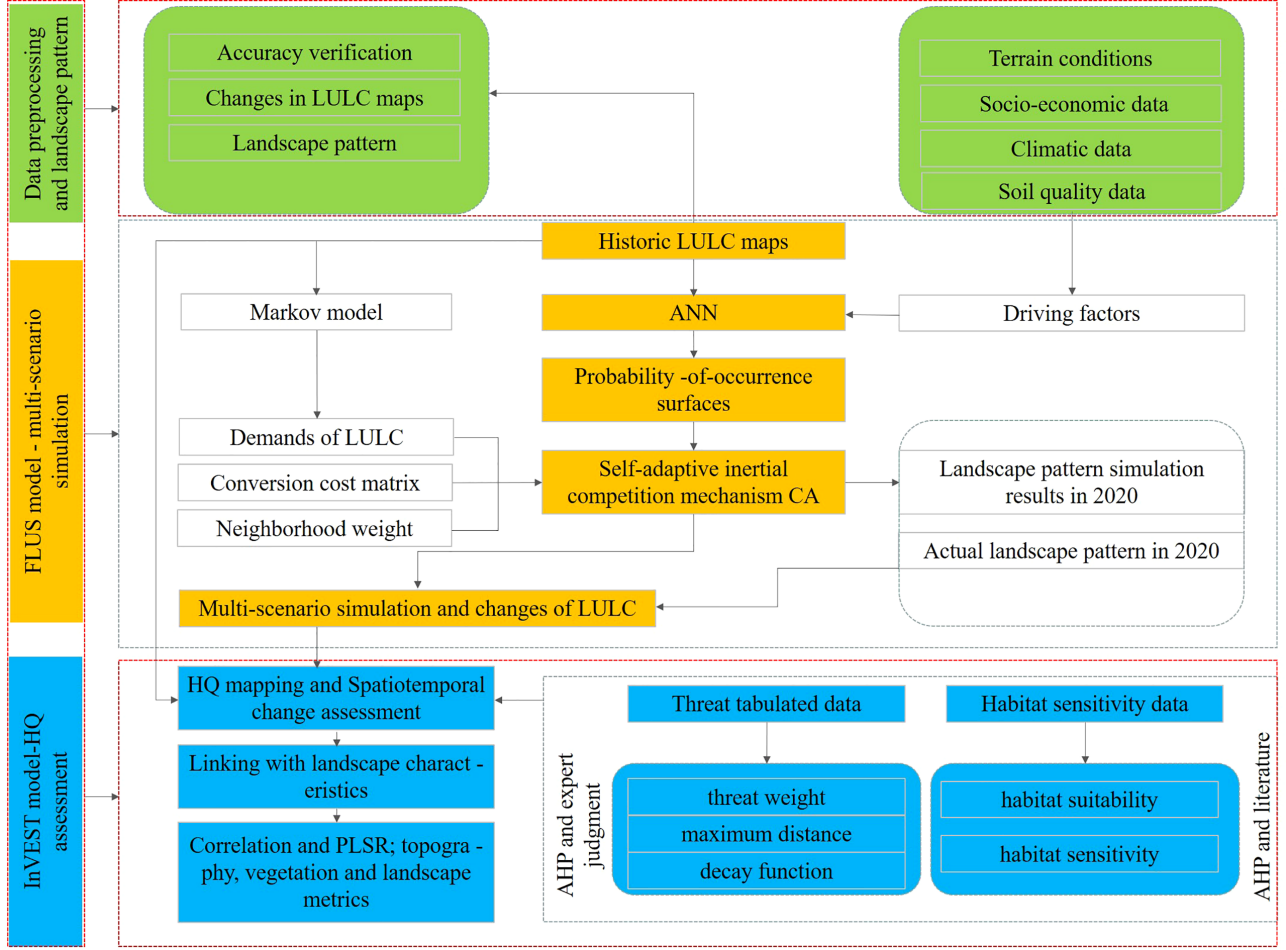
The research flow diagram.

The data used in the research including historic (2000, 2010, and 2020) and predicted (2030 and 2040) LULC maps (agriculture, forest, grassland, wetland, settlement and other), terrain conditions (Digital Elevation Model [DEM] and slope), socio-economic data (population [POP], urbanization rate [UR], GDP), climate data (temperature [tem] and precipitation [pre]) and soil quality (electrical conductivity [EC], acidity and alkalinity [pH], organic carbon [OC], and soil moisture [SM]). All the spatial datasets have been resampled to the same resolution of 0.3 km to complete the HQ multi-scenario simulation and prediction (**Supplementary Table 1**). Two sets of raster data (threat maps and LULC change maps) were processed by arcgis software (v 10.3) as inputs to the InVEST HQ model (**Figure 2**).

### 2.3 Scenario setting

Multi-scenario simulations were made of the LULC changes, which will help to improve protection measures, support policy decisions, and promote proper land use (Liu et al., 2017; Nehzak et al., 2022). Four scenarios were assumed based on the TRB’s regional climate changes and different socio-economic developments. They are the natural development scenario (NDS), the economic development scenario (EDS), the ecological priority scenario (EPS), and the harmonious development scenario (HDS). The NDS assumes the development trend remains unchanged (i.e., the historical development trend is used as the future simulation trend). In this study, the change trend of LULC in 2030-2040 is the same as the change trend that occurred in 2010-2020. The EDS assumes that the socioe-conomic benefits are maximized, according to the “rural revitalization strategy”. Under EDS, the urbanization process will be accelerated, and other LULC types will be further transformed into construction land. The probability of converting agriculture, grassland, wetland and other land types to settlement increased by 25%, and settlement remained unchanged. Compared to the EDS, the EPS is constructed to maximize ecological benefits. Under EPS, the probability of ecological land encroachment will be reduced by the promotion of ecosystem conservation and restoration policies. The probability of converting agriculture, grassland, wetland and other land types to settlement decreased by 50%, agricultural and settlement lands were transferred to forest, and grassland increased by 50%. Forestland remained unchanged. Finally, the HDS is a sustainable development mode, based on the above scenarios. The HDS assumes a 25% decrease in the probability of converting agriculture, grassland, wetland and other to settlement, while agricultural and settlement land is transferred to forest increases by 25%. Additionally, under this scenario, grassland increases by 25% and forestland remains unchanged.

### 2.4 Predicting the demands for LULC

The Markov chain was used to predict land demand under the four mentioned scenarios (NDS, EDS, EPS, HDS) for the years 2030 to 2040. The model is determined by the number of states and the probability of change between states, which helps to estimate LULC change probability from one state to another and to project future spatial changes (Mokarram et al., 2021). The specific process is as follows. The model calibration is performed using the 2020 LULC map and the transition probability from 2000 to 2010 to simulate the 2020 LULC map, while the actual classified image of 2020 is used to validate the simulated image accuracy. If the Markov chain prediction reaches a satisfactory level (Kappa coefficient greater than 0.75), a transition matrix derived from LULC data in 2010 and 2020 will be used to project land demand under different scenarios for the years 2030 and 2040.

### 2.5 FLUS model and parameterization

This research uses the FLUS model (v.2.4) to simulate future multi-scenario LULC patterns. Initially, training and estimating the probability-of-occurrence by considering 10 driving factors (DEM, slope, pop, GDP, tem, pre, EC, pH, OC, SM) and land use data (2010). Then, adjusting the land use probability-of-occurrence by taking into account land demand, neighbourhood influence (ranging from 0 to 1; the smaller the value, the weaker the expansion ability, and vice versa) and conversion cost (0 means no conversion, 1 represents conversion is allowed). Finaly, using the figure of merit (FOM, Eq.(A.1)) and kappa coefficient (Eq.(A.2)) to verify the simulation accuracy (Liu et al., 2022).


$$TProb_{p,k}^{t}=sp(p,k)\times \Omega_{p,k}^{t}\times Inertia_{k}^{t}\times (1-sc_{c\to k})$$

where *TProb^t^_p,k_* refers to the combined probability of raster cell *p* for conversion from the initial LULC type to the target LULC type *k* at iteration time *t*; *sp*(*p,k*), Inertia*^t^_k_*, and Ω*^t^_p,k_* are the probability-of-occurrence (Eq.(B1-B2)), inertia coefficient (Eq.(B3-B5)), and neighborhood effects (Eq.(B6)), respectively. and *sc_c→k_* refers to the conversion cost from the initial LULC type *c* to the target LULC type *k* (Liu et al., 2017; Liang et al., 2018).

### 2.6 InVEST habitat quality model

InVEST HQ module is a mature and effective tool to assess HQ by inputting LULC maps and threat factors. The present research used the HQ module (version 3.9.0), with the primary task aiming to identify threats to the habitat (Sharp et al., 2018). Based on expert knowledge, the InVEST user manual, and similar literature on the TRB (He et al., 2022), we evaluated the impacts of different threats (e.g., agriculture, settlement) on HQ. Using the Analytic Hierarchy Process (AHP) tool, we determined the weight of the threats and habitat sensitivity (Hou et al., 2021; Yohannes et al., 2021).

HQ was assessed for 2000, 2010, 2020 LULC and four different scenarios (NDS, EDS, EPS, HDS) using the InVEST HQ module. All input data (threats, maximum distance, weight and habitat suitability) used to simulate HQ are listed in **Table 1**. The value of HQ ranges from 0 (non-habitat quality) to 1 (maximum suitability habitat quality) (Mengist et al., 2021; Yohannes et al., 2021). In this study, the HQ was divided into five equal classes, they are poor (0–0.2), low (0.2–0.4), moderate (0.4–0.6), good (0.6–0.8), and high (0.8–1.0), respectively. Habitat degradation degree is also an indicator of HQ and the greater the value, the higher the degradation degree. The habitat degradation degree was divided into five classes (severe: > 0.078, moderate-severe: 0.044-0.078 moderate: 0.021–0.044, slight: 0.006–0.021, and no: 0–0.006).

**Table 1:** The important date used to Input habitat quality model.

	Threats	Maximumdistance (km)	Weight	LULC considered as habitat			
				Forest	Grassland	Wetland	Other
Habitat suitability				1	0.4	1	0.5
Sensitivity	Settlement	8	1	0.6	0.6	0.7	0.8
	Agriculture	1	0.5	0.4	0.5	0.4	0.3

The model is dependent on either the exponential or linear distance decay function to describe the influence of the threat factors (Sharp et al., 2018).


$$i_{rxy}=1-\left(\frac{d_{xy}}{d_{r\max}}\right)\;if\;linear$$

$$i_{rxy}=\exp\left(-\left(\frac{2.99}{d_{r\max}}\right)d_{xy}\right)\;if\;exponential$$

where *d_xy_* is the distance between raster cells *x* and *y*, and *d_rmax_* refers to the maximum threat distance of r.

The habitat degradation degree (*D_xj_*) of raster cell *x* in LULC type *j* can be expressed as (Sharp et al., 2018):


$$D_{xj}=\sum_{r=1}^{R}\sum_{y=1}^{yr}\left(\frac{w_{r}}{\sum_{r=1}^{R}w_{r}}\right)r_{y}i_{rxy}\beta_{x}S_{jr}$$

where *r* refers to the number of threats; *y_r_* refers to the number of grid cells of *r’s* map; and *r_y_* refers to the threat intensity of raster cell *y*. The *i_rxy_* refers to the impact of *r_y_* on the stress level of *x*; *ω_r_* refers to the weight constant; and *β_x_* refers to the accessibility level. The *S_jr_* refers to the relative sensitivity of *j* to *r*.

The HQ index (*Q_xj_*) of grid *x* in LULC type *j* is calculated as follows (Sharp et al., 2018):


$$Q_{xj}=H_{j}\left(1-\left(\frac{D_{xj}^{z}}{D\frac{Z}{xj}+k^{Z}}\right)\right)$$

where *H_j_* refers to the habitat suitability of LULC type *j*, the range is 0–1(0 means no habitat suitability, and 1 means highest habitat suitability); *z* and *k* are normalized constant(z=2.5), half-saturation constant(k=0.5), respectively.

### 2.7 Landscape pattern metrics

The landscape index condenses a large amount of landscape pattern information that reflects the characteristics of spatial configuration and structural composition (Guo et al., 2021; Shuangao et al., 2021; Yang, 2021). In striving to choose elements that are representative, simplified and common (Mengist et al., 2021), and by referring to relevant research results (Guo et al., 2021; Yang, 2021) and combining the actual situation of the study area, we choose aggregation index (AI), area-weighted mean fractal dimension index (FRAC_AM), mean patch area (AREA_MN), number of patches (NP), largest patch index (LPI), and percentage of landscape (PLAND). All of these indices were computed using the Fragstats software (v. 4.2.1).

### 2.8 Partial least squares regression (PLSR)

PLSR is a robust multivariate regression technique (Li et al., 2019) that combines the advantages of principal component, multiple linear regression, and correlation analysis (Xu et al., 2022). It not only provides a quantitative simulation of the complicated relationships between independent and dependent explained variables (Shawul et al., 2019; Yohannes et al., 2021), but can also easily process the data with high dimensionality and multicollinearity (Sun et al., 2021).

For PLSR, there are some useful model parameters, as follows. The goodness of prediction (Q^2^) and explained variation in the response (R^2^) were used to assess model performance (Shawul et al., 2019). The regression coefficients (Co) and variable importance in projection (VIP) indicate the influence direction and importance of independent variables for the dependent variables (Li et al., 2019). The weight (W) offers both the direction and the magnitude of the relationship between the independent and dependent variables (Shawul et al., 2019). Landscape metrics were independent variables and HQ was a dependent variable in this study. Both of them as inputs to the PLSR model, which was performed using SIMCA (v. 14.1). Origin 2022 was used to perform the statistical analysis of the dataset.

## 3 Results

### 3.1 LULC and dynamics in the TRB (2000-2020)

**Figure 3:**
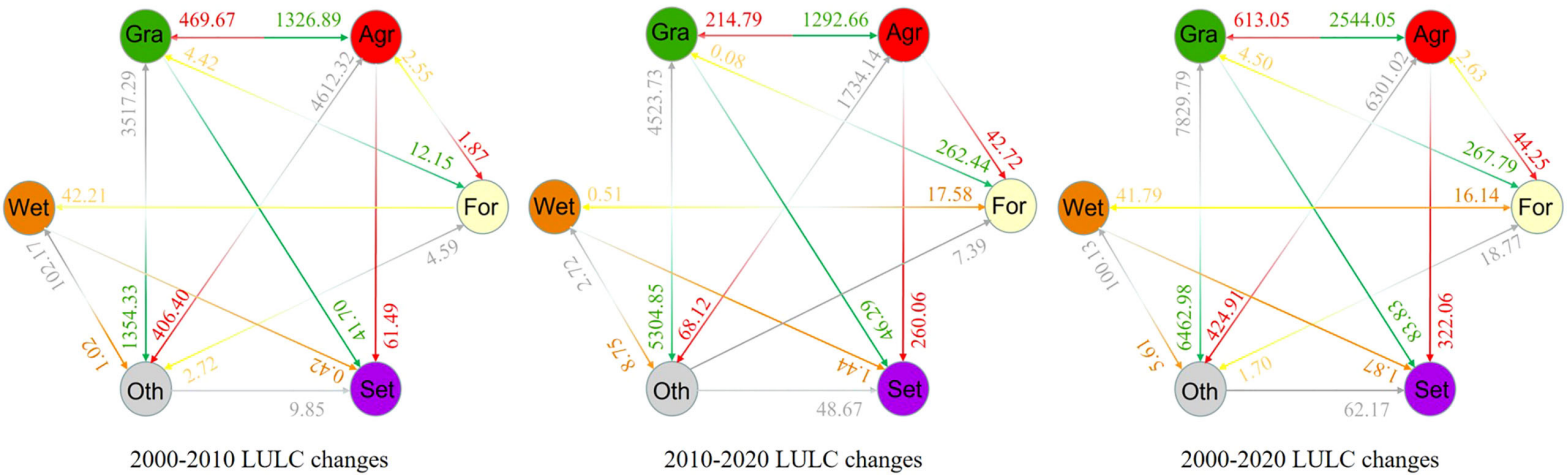
Transfer matrix of each LULC class for 2000-2010, 2010-2020, and 2000-2020. Gra, Agr, For, Set, and Wet refer to grassland, agricultural land, forestland, settlement, and wetland respectively. Whereas Oth refers to other type of LULC which include water, bare land, snow and ice. Different colors represent different LULC types, arrows represent the direction of LULC type transfer, and corresponding numbers represent the area of LULC transfer.

The LULC in TRB has changed dramatically over the past 20 years. Agriculture, forest, wetland and settlement areas have shown increasing trends, while grassland and other areas have rapidly decreased (**Figure 3**; **Supplementary Figures 1, 2**). Among these land use types, agricultural, forest and settlement areas expanded the most in the period under study, changing from 5.62%, 0.06%, and 0.02% in 2000 to 6.45%, 0.09%, and 0.07% in 2020, with an annual growth rate of 372.17, 14.82 and 23.50 km^2^, respectively. The increase in agricultural land is ascribed to the reduction in grassland (-2544.05 km^2^) and other land use types (-6301.02 km^2^). Population growth requires more food production, which in turn leads to an increase in agricultural area. In the agricultural area, 322.06 km^2^ was transformed into settlement, accounting for 68.5% of the increased area of settlement. This change may be caused by the encroachment of agricultural land around residential areas due to human activities. The increase in forest area is mainly due to the reduction of grassland area. It may be the climate change causes shrub encroachment in grassland. The increase in wetland area is mainly due to the reduction of Oth (including bare land, ice and snow), it can be seen that the wetlands are mainly located on the southern slope of the Tianshan Mountains, the upper reaches of the Kaidu River and the Bosten Lake Basin, which may be related to the rise in temperature and the increase in ice and snow melt water.

As well, the spatial variability has also changed significantly during 2000–2020. We found that the LULC changes mainly occurred around the Taklimakan Desert, such as along rivers and in oasis and piedmont meadow areas. While 0.75% of the LULC improved, 2.42% of it declined. For other land use types, most of the LULC was unchanged, including bare areas (desert), water bodies, and permanent snow and ice. **Figure 3** shows the annual change rate combined with the transfer matrix data information, illustrating further analysis of LULC changes during different phases of the study period.

### 3.2 Projected LULC changes under different scenarios (2030–2040)

The overall accuracy, kappa coefficient and FOM reached 96.31%, 91.85% and 3.4% in this study, respectively. This indicates the simulation results are reliable. A summary of the LULC changes and the spatial pattern under different scenarios (**Supplementary Figure 3**). As expected, the agriculture and settlement area will continue to increase in 2030 and 2040 for all scenarios (NDS, EDS, EPS, HDS), with an annual growth rate from 216.61 to 240.09 km^2^ and from 1.57 to 13.25 km^2^, respectively, which indicates a high degree of human interference. Other (including bare land, water body) is the land use type with the largest area reduction, which decline from -1970.96 to -2043.98 km^2^ per year, maybe it is used for development purposes that convert into agricultural land.

In the EDS, settlement experiences dramatic growth compared to the NDS, with an annual growth rate of 13.25 km^2^. This may be due to the acceleration of urbanization process, which need to increase settlement to accommodate economic and population growth. In the EPS, the area of forestland and grassland has expanded rapidly, which is anticipated in the ecological conservation and environmentally friendly development scenario. Furthermore, compared to the NDS, the growth of agricultural area has slowed down and the increase in grassland area has accelerated. Meanwhile, under the HDS, settlement and forest have moderately increased, this may be attributed to the trade-off between socio-economic and ecological benefits. The LULC changes in the NDS and EDS have similar spatial distribution characteristics, while the spatial distribution is similar in the EPS and HDS.

### 3.3 Association between HQ and landscape pattern metrics

**Figure 4:**
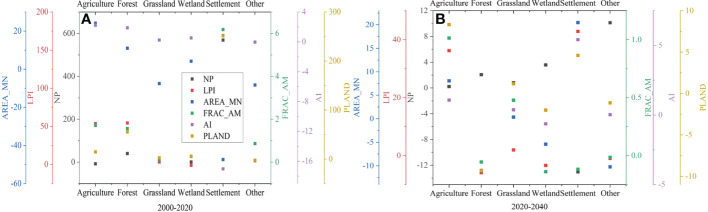
Percentage of landscape metrics changes of each LULC class for 2000-2020 **(A)** and 2020-2040 **(B)**. NP number of patches; LPI, largest patch index; AREA_MN, mean patch area; FRAC_AM, area weighted mean fractal dimension index; AI, aggregation index; PLAND, percentage of landscape.

The landscape structure in the TRB has experienced significant changes over the past 20 years (**Figure 4A**). The LPI, NP and PLAND increased for all LULC categories, except for wetland (-0.90%), agriculture (-7.56%) and other (-2.07%), respectively. The increase in these metrics indicates an expansion of patch areal sizes and fragmentation. The change in AREA_MN has increased in agriculture (24.19%), forestland (11.04%) and wetland (4.24%), which is due to the gradual accumulation of patches, resulting in the increase of patch area. Conversely, it has decreased for grassland (-7.49%), settlement (-47.36%) and other (-8.26%), which indicates patch fragmentation. Moreover, the patch AI increased for all LULC categories, except for settlement (-17.02%) and other (-0.03%), and the patch FRAC_AM increased for all LULC categories. The value of most of the landscape metrics for the projected LULC is less than for the previous period (2000-2020) (**Figure 4B**). This indicates that the expansion of patch areal sizes and fragmentation has slowed down, and that most of the patch shape boundaries have become simpler.

### 3.4 Spatio-temporal distribution of HQ in the TRB

#### 3.4.1 HQ assessment (2000–2020)

**Figure 5:**
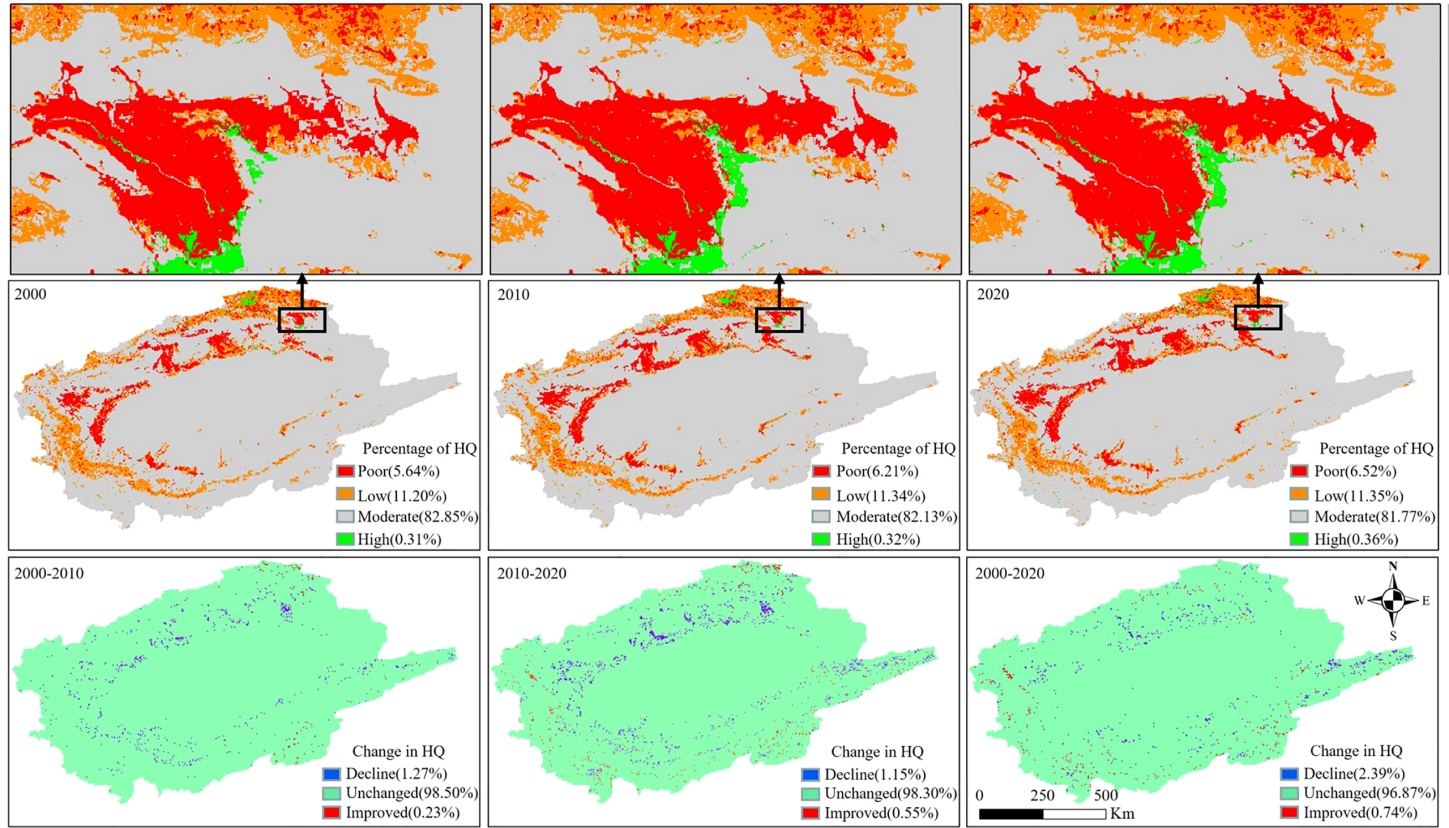
Spatial distribution and changes of habitat quality (HQ) level, and percentage share from 2000 to 2020.

The HQ in the TRB was dominated by moderate and low grades, with the average HQ decreasing from 0.449 to 0.444 during 2000–2020. This decline points to an overall deterioration of the HQ. Specifically, the area of high HQ and poor HQ increased from 0.31% to 0.36% and 5.64% to 6.52%, respectively (**Figure 5**). This showed that the watershed has experienced HQ degradation, only slightly improved over the past 20 years. The high HQ is mainly distributed in forested areas and wetland, with less affected by human interference. The poor HQ is mainly distributed in agriculture and settlement, which clearly indicates that the expansion of agricultural and settlement land use has threatened HQ.

#### 3.4.2 Prediction of HQ under different scenarios (2030–2040)

**Figure 6:**
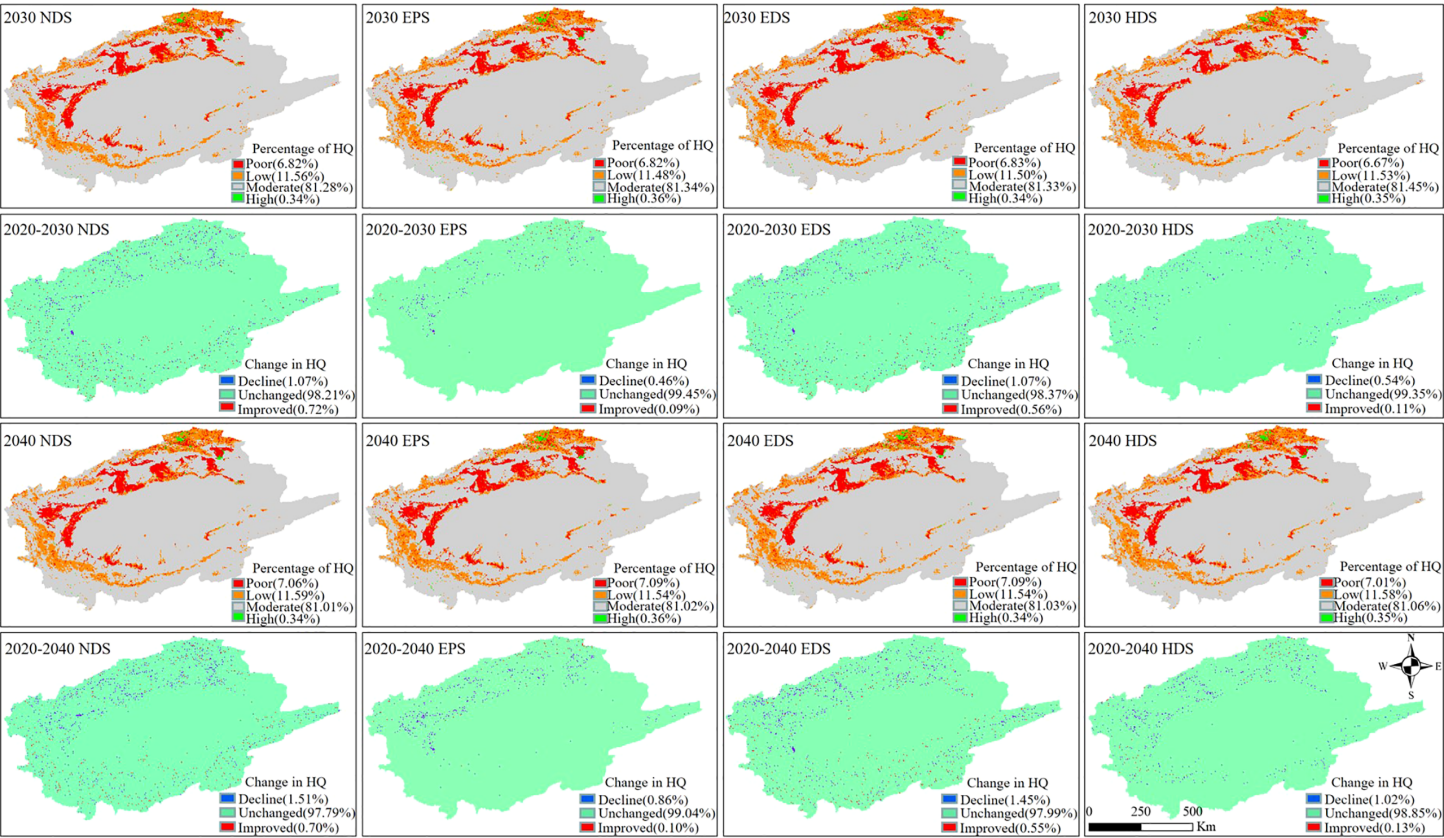
Spatial distribution, percentage share and changes of each projected habitat quality under different scenarios. NDS, natural development scenario; EDS, economic development scenario; EPS, ecological priority scenario; HDS, harmonious development scenario.

The spatial pattern of HQ in 2030–2040 is basically similar to that of the previous decades (2000–2020) under different scenarios (**Figure 6**). High HQ is mainly prevalent in forest and wetland, moderate HQ is widely distributed throughout the region, low HQ primarily occurs in grassland, and poor HQ is mostly found in agricultural and settlement areas. More than 97.79% of the habitat is unchanged in all scenarios, but only 0.70% has improved. This shows that the HQ is in a continuous decline. Based on the average value of the HQ in each scenario, the highest HQ value appeared in HDS and the lowest HQ value in EDS. These results show that giving priority to the development of the economy (i.e., the expansion of agriculture and settlement) will seriously threaten HQ. Further, the results also indicate that harmonious development is the preferred choice to maintain the highest level of HQ and socio-economic benefits.

### 3.5 Habitat degradation analysis

**Figure 7:**
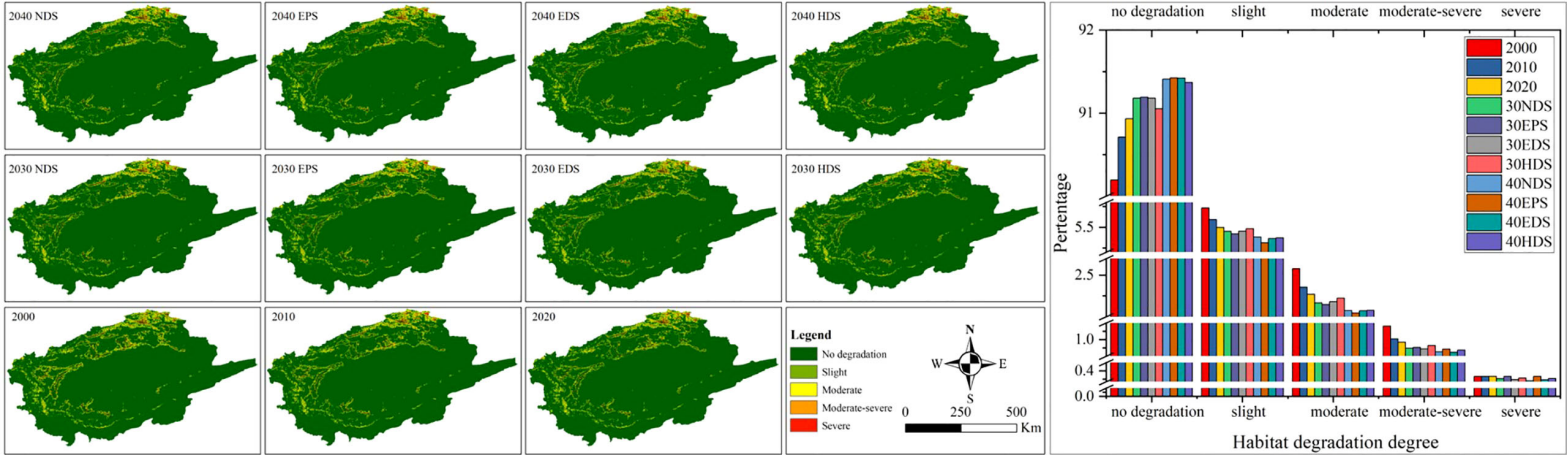
Spatial distribution and percentage share of habitat degradation degree in the TRB from 2000 to 2040 under different scenarios. TRB, Tarim River Basin; NDS, natural development scenario; EDS, economic development scenario; EPS, ecological priority scenario; HDS, harmonious development scenario.

**Figure 7** shows the habitat degradation degree in TRB from 2000 to 2040. The study area is dominated by non-degraded habitats, accounting for more than 90% of the total area. The habitat degradation areas with slight, moderate and moderately severe decreased from 5.73% to 5.31% (EPS), from 2.58% to 2.04% (EPS) and from 1.16% to 0.85% (EDS), respectively. This indicates a slight improvement in habitat degradation and EPS is the preferred model for maintaining a high-quality habitat.

From the perspective of spatial distribution in spatial distribution, moderate-to-severe habitat degradation is distributed in agricultural and settlement areas, whereas slight-to-no-degradation is located around forest, grassland, wetland, and other areas. One possible reason for this is that a natural habitat has only limited human activities, whereas a non-natural habitat designates a high degree of anthropogenic interference. This indicates that anthropogenic drivers (the expansion of oasis agriculture, rapid population growth, and resettlement campaigns) are the main factors leading to habitat degradation.

### 3.6 Factors influencing the spatio-temporal patterns of habitat quality

**Figure 8:**
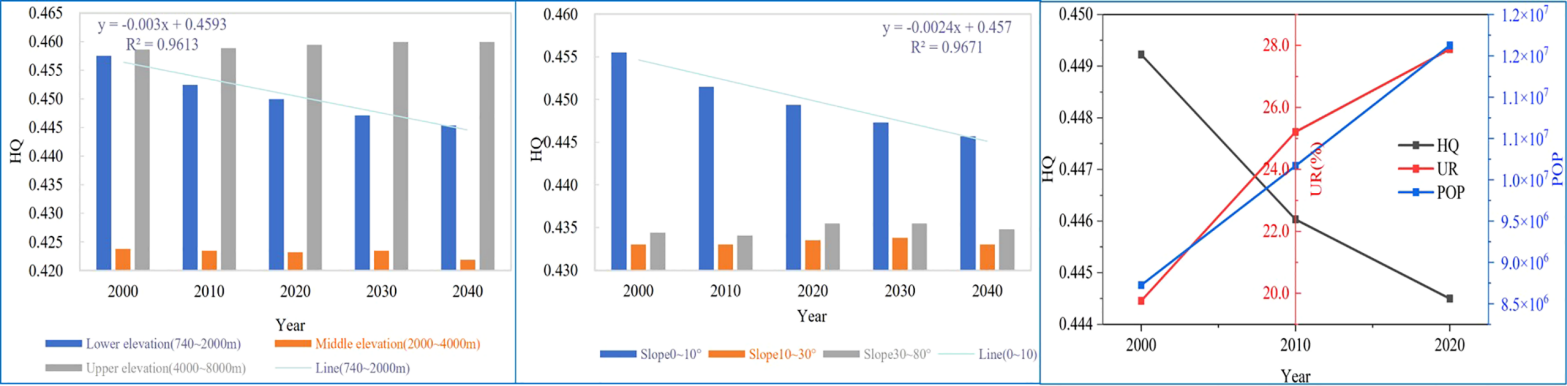
Changes of habitat quality (HQ) with slope, elevation and anthropogenic factors. UR, urbanization rate; POP, population.

We found that both natural and anthropogenic factors influenced the HQ. Our analysis showed that although HQ was highest in 2000 in the lower elevations (740-2000m) and on sloped land (<10°), it declined over time (**Figure 8**). In addition, HQ was high compared to slopes (>10°) and middle elevations (2000-4000m), but lower than the upper elevation (>4000m). Furthermore, we found that HQ showed a strong negative correlation with agriculture (p<0.01), urbanization rate (p<0.01), and settlement (p<0.05), while it had a significant positive correlation with the category of ‘other’ (p<0.05) (**Table 2**). This correlation is likely related to the expansion of agriculture and settlement, as other was occupied by agriculture and grassland.

**Table 2:** Habitat quality and its correlation with influencing factors.

Factors									
	Agr	For	Gra	Wet	Set	Oth	Deg	UR	POP
HQ	-1.000^**^	-0.735	-0804	-0.437	-0.888^*^	0.879^*^	0.868	-1.000^**^	-0.979

Agr, agricultural land; For, forestland; Gra, grassland; Wet, wetland; Set, settlement. Whereas Oth refers to other types of LULC which include water, bare land, snow and ice. UR, urbanization rate; POP, population. *correlation is significant at 0.05 level, **correlation is significant at 0.01 level.

3.7. Contribution of landscape structural change to HQ

**Table 3** summarized the PLSR analysis for HQ. The first two components of all X variables (except wetland) cumulatively explain more than 92.5% of HQ variability, while the cumulative variance for the first two components of all Y variables explain more than 93.6%. Furthermore, the Q² cumulated (Q²_cum_) of all landscape variables explained more than 0.826 (except for wetland and settlement).

**Table 3:** Summary of the PLSR model output with respect to habitat quality.

	Response variable	Component	R^2^X	R^2^X(cum)	R^2^Y	R^2^Y(cum)	Q^2^	Q^2^(cum)
**Agr**	Habitat quality	1	0.883	0.883	0.963	0.963	0.943	0.943
		2	0.104	**0.986**	0.0155	**0.978**	0.172	**0.952**
**For**	Habitat quality	1	0.652	0.652	0.781	0.781	0.595	0.595
		2	0.305	**0.957**	0.207	**0.988**	0.906	**0.962**
**Gra**	Habitat quality	1	0.735	0.735	0.963	0.963	0.888	0.888
		2	0.191	**0.925**	0.0163	**0.98**	0.203	**0.911**
**Wet**	Habitat quality	1	0.363	0.363	0.968	0.968	0.439	0.439
		2	0.49	0.852	0.0226	**0.991**	0.59	0.77
**Set**	Habitat quality	1	0.722	0.722	0.923	0.923	0.813	0.813
		2	0.263	**0.985**	0.013	**0.936**	-0.15	0.794
**Oth**	Habitat quality	1	0.666	0.666	0.984	0.984	0.842	0.842
		2	0.281	**0.947**	0.0012	**0.985**	-0.156	0.826

Agr, agricultural land; For, forestland; Gra, grassland; Wet, wetland; Set, settlement. Whereas Oth refers to other types of LULC which include water, bare land, snow and ice. Bold face values indicate that the PLSR model is regarded as excellent.

**Figure 9:**
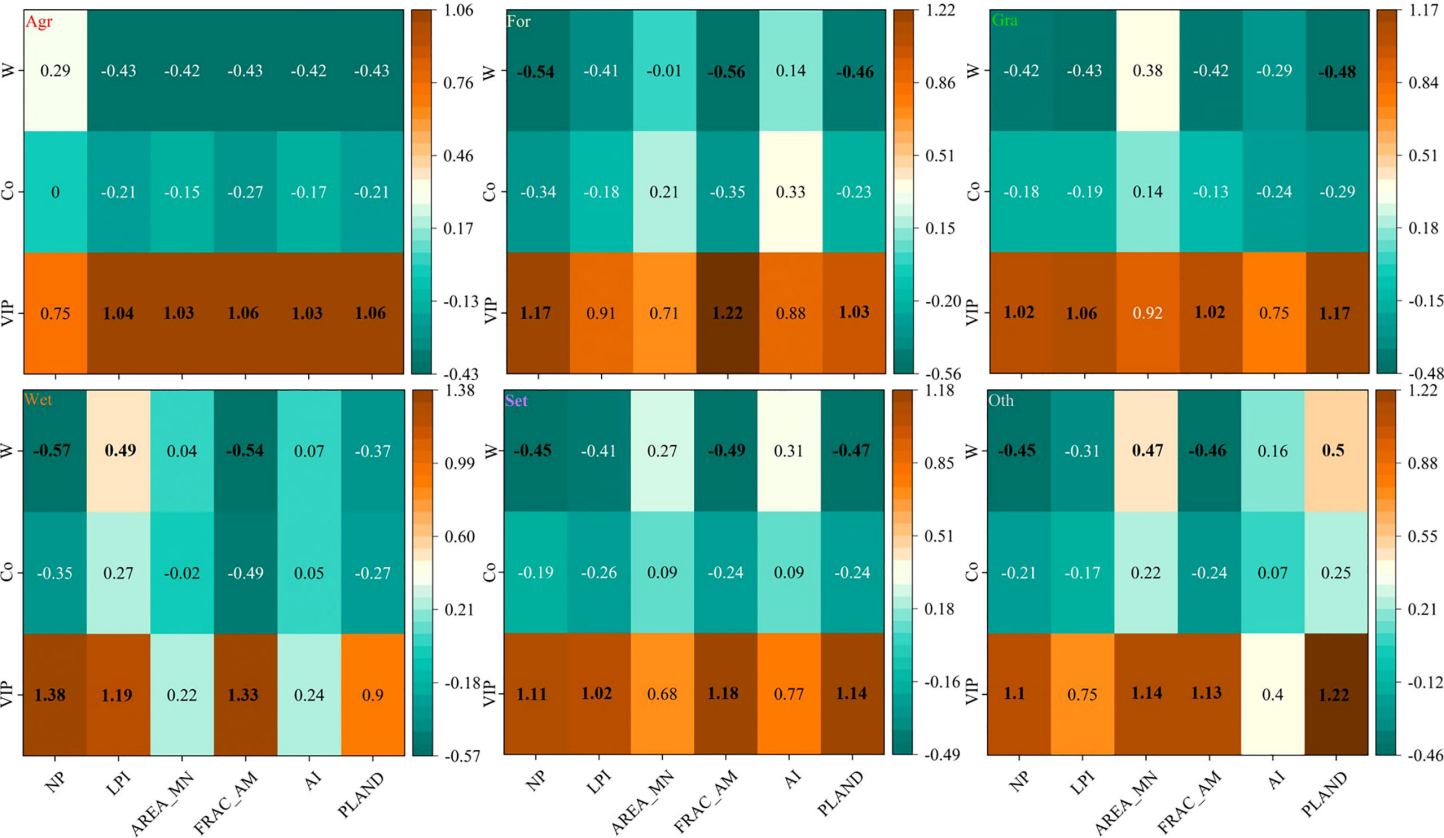
PLSR variable importance and weights of the first component and regression coefficient for HQ model. Co, regression coefficients; VIP, variable importance in projection; W, weight; NP, number of patches; LPI, largest patch index; AREA_MN, mean patch area; FRAC_AM, area-weighted mean fractal dimension index; AI, aggregation index; PLAND, percentage of landscape; Agr, agricultural land; For, forestland; Gra, grassland; Wet, wetland; Set,settlement. Whereas Oth refers to other types of LULC which include water, bare land, snow and ice. Bold face, values indicate (w^2^ > 0.2) the importance of predictor variables.

In this study, VIP, Co and weights were used to explore the relative influence of each landscape structural variable on HQ. Moreover, the bold face values showed that the PLSR component is more important to the response variables (**Figure 9**). For changes in HQ, the highest VIP value for NP was obtained by wetland (VIP =1.33; w = -0.57; Co =-0.55), followed by forestland (VIP =1.17; w = -0.54; Co =-0.34). Similarly, for LPI, AREA_MN, FRAC_AM, AI and PLAND, the highest VIP value was obtained by wetland (VIP =1.19; w = 0.49; Co =0.27), other (VIP =1.14; w = 0.47; Co =0.22), wetland (VIP =1.33; w = -0.54; Co =-0.49), agriculture (VIP =1. 03; w = -0.42; Co =-0.17) and other (VIP =1.22; w = 0.50; Co =0.25), respectively.

## 4 Discussion

### 4.1 Spatio-temporal patterns of HQ

Assessing the spatio-temporal changes of HQ in TRB is helpful for developing conservation strategies and harmonizing the relationship between ecological balance and economic development. It also helps to improve ecological integrity. Furthermore, by identifying areas of poor and low HQ, we can help to improve biodiversity. In this study, the poorest HQ was concentrated in agricultural and settlement areas with the maximum degree of human interference. The expansion of oasis agriculture and settlements, which caused habitat fragmentation and a decline in native habitat (grassland).

The reason may be the population growth requires more food production and residence, which in turn leads to an increase in agricultural and settlement area. According to the urban system planning (2012-2030) of Xinjiang, by 2030, the urbanization rate will be 65% - 70%. This means that more residences are needed, which will inevitably lead to the transformation of other land use types to residences, thus leading to the degradation of habitat quality. Another reason is the government’s resettlement campaigns. According to these resettlement policies, a new socialist countryside needs to be built, fueled by a “rural revitalization strategy”. Within this process, urbanization will be further accelerated and other LULC types will be transformed into construction land. The policy also anticipates the abandonment of old homesteads, resulting in the further deterioration of HQ.

In contrast, the highest HQ was in forest and wetland areas, due mainly to the low level of anthropogenic interference in those locales. It is also related to the implementation of the “forest law of the People’s Republic of China” in Xinjiang Uygur Autonomous Region in 2001. According to this policy, government departments must halt forest destruction by designating closed cultivation areas and closed cultivation periods. As well, they had to designate protected areas for natural forests, implement key protection, and strictly prohibit logging. In addition, it is also related to climate change. In recent years, the precipitation and the melting water of ice and snow in mountain areas has increased, which has restored the forest land and wetlands

### 4.2 Spatio-temporal patterns of HQ degradation

This research confirms that while habitat degradation has gradually declined over the whole study period, the highest and most persistent degradation is clustered in agriculture and settlement areas. These findings are similar to those found elsewhere (Zhu et al., 2020; Mengist et al., 2021). Natural habitats (e.g., grassland, other, and forest), for instance, that are situated close to agricultural areas will convert into agricultural land with high probability. In addition, smaller patches situated adjacent to agricultural areas may also convert to agricultural lands. This, in turn, results in habitat fragmentation and loss, which could lead to HQ degradation. In all scenarios, the EPS is the preferred model for maintaining high-quality habitat, while giving priority to the development of the social economy through the expansion of agricultural and settlement lands. However, unmanaged expansion may seriously threaten the HQ of those areas.

Additionally, the spatial changes in habitat degradation indicate that the closer a land type is to human-modified areas (agricultural and settlement), the more likely it is to be degraded. This confirms that there is a close connection between habitat degradation and human activities. Therefore, the concept of natural conservation should be emphasized in the process of social and economic development, and it is necessary to implement ecological restoration and mitigate habitat degradation. The assessment and projection of HQ and degradation have enabled people to have a more comprehensive understanding of the ecological environment and human impacts on the TRB. Hence, mapping the spatio-temporal distribution of HQ in the TRB would be a valuable contribution that could offer a scientific basis for decision-makers to formulate practical conservation strategies that are conducive to preventing the deterioration of regional HQ.

### 4.3 Factors that threaten HQ

Although both natural and anthropogenic factors are generally considered to be the main factors affecting ecosystems, anthropogenic activities have had an enormously negative impact on Earth since the Industrial Revolution. This can be seen in the degradation of native habitats, loss of biodiversity, vegetation degradation, species extinction, and ecological degradation. The dawn of the proposed *Anthropocene* period has seen human activities dramatically changing Earth’s systems, which in turn will aggravate ecosystem deterioration and habitat degradation. Currently, most countries are facing severe challenges to their ecological environments due to human activities such as industrialization, agriculture, and urbanization. Human activities are the main factors driving LULC changes that in turn result in HQ decline.

For instance, this study concluded that HQ showed a strong negative correlation with agriculture (p<0.01) and settlement (p<0.05), these results are similar to those of other studies elsewhere (Zhu et al., 2020; Tang et al., 2020; Mengist et al., 2021; Yohannes et al., 2021). The ecological restoration of the lower reaches of Tarim River has achieved remarkable results since the implementation of the ecological water conveyance project in 2000, bringing huge ecological, social and economic benefits to the local area. At the same time, there were differences in HQ at different slopes and altitudes. For instance, the HQ was generally better in low slope and high elevation than in steep slope and low elevation, respectively. This is possible due to lower slopes being more suitable for plant growth but less suitable for human activities. Again, these findings are similar to those in other studies, such as the research conducted by (He et al., 2022).

In the PLSR modeling, if the R^2^ > 0.5, it is considered to provide good predictions (Li et al., 2019); if Q²_cum_ is greater than 0.8, it is regarded as excellent (Yohannes et al., 2021). In this study, the Q²_cum_ > 0.826 (except for wetland and settlement) and R^2^_cum_ is greater than 92.5% (except for wetland), which indicates that the PLSR model was excellent for predicting HQ. Furthermore, the VIP, Co and W are important parameters for predicting independent and dependent variables. Generally, VIP values greater than 0.8, greater than 1 and less than 0.5 mean the importance is significant, the most relevant, and weak (meaningless), respectively (Shawul et al., 2019; Gan et al., 2020). The squares of the W value that are > 0.2 indicate that the independent variable is more important for the dependent variable (Shawul et al., 2019).

### 4.4 Uncertainty of models and future outlook

Models are effective and reproducible tools that can be used for analysing both the causes and consequences of a situation (Liu et al., 2017). However, modelling is also subjected to uncertainty. The FLUS model compensates for the shortcomings of traditional land use simulations and provides an effective way for projecting LULC changes. Nevertheless, the FLUS model also has a few shortcomings. For example, it cannot simulate the patch, which needs to be improved by adding a patch (Liu et al., 2017). Another limitation is that the conversion rules (e.g., the conversion costs) are assumed to be invariant, but these rules may change in the real world, due to shifts in development policy that may alter the current status (e.g., facilitate or limit LULC changes) (Miksa et al., 2020). Moreover, the future data were based on predictions, which are also uncertain. Therefore, each scenario’s outcomes in this study should provide only guidelines to managers and decision-makers, and extrapolations should be carried out with caution (Gomes et al., 2021).

Using the InVEST HQ module to assess HQ has its advantages compared to other ecological tools (CA, ANN, Markov, logistic-CA, ANN-CA, and CLUE-S). However, it also has some shortcomings when assessing HQ. For instance, the parameters of threat factors and habitat sensitivity were confirmed based on expert knowledge; thus, subjectivity was unavoidable, although we used AHP to minimize possible errors and to add objectivity (Yohannes et al., 2021; He et al., 2022). More effort needs to be invested in this model to improve it for future work (Liu et al., 2017).

### 4.5 Policy implications for ecologically fragile areas

The spatio-temporal assessment of HQ is helpful for decision-makers to use in making viable plans for the future. Moreover, exploring the impact of LULC landscape changes on HQ is significant for maintaining biodiversity and the integrity of native habitats. In this study, the ratios of low/poor HQ areas are increasing, and HQ threatening factors are impacting changes in LULC landscape due to anthropogenic interference. As reflected by LULC type, forestland and wetland, which have integrated ecological systems, had the highest HQ, while the habitat degradation degree is greatest in the agricultural and settlement areas. Consequently, good HQ relies on the reasonable and effective control of the transformation of LULC types (Zhang et al., 2020).

Implementing the necessary policies is an effective way to mitigate habitat degradation. In recent years, the state and local government have made great efforts to restore ecological problems. For example, the ecological water conveyance project in the lower reaches of the Tarim River for ecological restoration and environmental conservation has achieved initial results, which is of great significance for improving habitat quality. The “Master Plan for Major Projects of National Important Ecosystem Protection and Restoration (2021-2035)” and the “Master Plan for Major Projects of xinjiang Important Ecosystem Protection and Restoration (2021-2035)” pointed out that major projects for ecological protection and restoration in the Tarim River Basin should be implemented, of which the state invested 493.8 million yuan for ecological security construction in Xinjiang, including the Tarim River Basin (Hotian River Basin, Tianshan Forest and Grassland Protection, Yili River Valley), etc. It is planned to build 11693.33 hm^2^ of arbor forest, 6046.67 hm^2^ of shrub forest, 7933.337 hm^2^ of degraded forest restoration, 22133.34 hm^2^ of mountain closure for forest cultivation, 17266.68 hm^2^ of artificial grass planting, 27266.68 hm^2^ of enclosure for cultivation, 59333.36 hm^2^ of grassland improvement, etc. This will greatly promote the habitat improvement of Tarim River and other basins

Furthermore, assessing changes in HQ and the impact of human activities under different scenarios can help to harmonize economic development and ecosystem balance. In this study, EDS agriculture and settlement expansion occurred at the expense of decreases in natural habitat, which led to the degradation of HQ. EPS aims to protect natural habitats and prevent degradation, which is conducive to improving the ratios of high HQ. Compared to EDS and EPS, HDS not only improves HQ, but also contributes to economic development. Therefore, EPS and HDS are crucial for systematic nature conservation planning.

Overall, this study can provide basic information for decision-makers to formulate conservation policies. It can also assist in the identification of habitats that need protection in order to alleviate ecological degradation. In addition, the findings in this research can help the relevant authorities to harmonize the relationship between economic development and ecological conservation.

## 5 Conclusions

Assessing the impact of LULC landscape changes on HQ under multiple scenarios is crucial for planning regional management interventions. The results of this research showed that the TRB has had a declining trend (from 0.449 to 0.444) in HQ since 2000. The poorest HQ was concentrated in agricultural and settlement areas, while the highest HQ was in forestland and wetland. Correspondingly, the degree of habitat degradation in agricultural and settlement areas was higher than in native habitats, with the expansion of oasis agriculture and settlements causing habitat fragmentation and a decline in native habitat. Thus, the TRB needs adequate strategies to protect natural habitats in the future.

This study found that human activities are the main factors driving LULC changes that in turn result in HQ decline. However, NP, FRAC_AM, PLAND and LPI were also important contributors to HQ change. Specifically, NP and FRAC_AM were important for natural habitats, while PLAND and LPI were important for anthropogenic habitats. In all scenarios, both EPS and HDS were shown to be ideal models for maintaining high-quality habitats. This study can provide basic information for decision-makers to help formulate conservation policies for natural habitats, while at the same time helping to harmonize the relationship between economic development and ecological conservation by designing effective and sustainable management plans.

## Data availability statement

The original contributions presented in the study are included in the article/**Supplementary Material**. Further inquiries can be directed to the corresponding author.

## Author contributions

TZ: Formal analysis, Methodology, Writing–original draft. YC: Conceptualization, Supervision, Funding acquisition. All authors contributed to the article and approved the submitted version.

## Funding

This work was supported by the Natural Science Foundation of Xinjiang (2021D01D01), the Strategic Priority Research Program of Chinese Academy of Sciences (XDA20100303) and the Key Research Program of the Chinese Academy of Sciences (ZDRWZS-2019-3).

## Conflict of interest

The authors declare that the research was conducted in the absence of any commercial or financial relationships that could be construed as a potential conflict of interest.

## Publisher’s note

All claims expressed in this article are solely those of the authors and do not necessarily represent those of their affiliated organizations, or those of the publisher, the editors and the reviewers. Any product that may be evaluated in this article, or claim that may be made by its manufacturer, is not guaranteed or endorsed by the publisher.
