# Supplementary material for: The effects of landscape change on habitat quality in arid desert areas based on future scenarios: Tarim River Basin as a case study
Source: Front Plant Sci. 2022 Oct 25;13:1031859. doi: 10.3389/fpls.2022.1031859 (PMC9642338; doi:10.3389/fpls.2022.1031859)
Supplement: Supplementary file 1 [file DataSheet_1.doc]

The effects of landscape change on habitat quality in arid desert areas based on future scenarios: Tarim River Basin as a case study

Tianju Zhang^1,2^, Yaning Chen^1*^ , Sikandar Ali^1,2^ and Xigang Liu

^1^State Key Laboratory of Desert and Oasis Ecology, Xinjiang Institute of Ecology and Geography, Chinese Academy of Sciences, Urumqi 830011, China

^2^University of Chinese Academy of Sciences, Beijing, 100049, China

Appendices

Table 1 List of data used in this study

| Category | Date | resolution | Date resource |  |
| --- | --- | --- | --- | --- |
| Land use land cover | LULC date | 300m | ESA(<http://maps.elie.ucl.ac.be/CCI/viewer/>); C3S (http://cds.climate.copernicus.eu/) |  |
| Terrain | DEM | 1Km | NOAA (https://www.ngdc.noaa.gov/mgg) |  |
|  | Slope | 1Km | Calculated from DEM |  |
| soil | the electrical conductivity (EC) | 1Km | FAO (<http://www.fao.org/soils-portal/soil-survey/soil-maps-and-data>base s) |  |
|  | the acidity and alkalinity (pH) | 1Km | FAO (<http://www.fao.org/soils-portal/soil-survey/soil-maps-and-database> s) |  |
|  | the organic carbon (OC) | 1Km | FAO (<http://www.fao.org/soils-portal/soil-survey/soil-maps-and-database> s) |  |
|  | soil moisture  (SM) | 0.05° | ((http://data.tpdc.ac.cn)) |  |
| Socio-economic data | population (POP) | 1Km | WorldPop (https://www.worldpop.org/);Statistical yearbook of Xinjiang (http://www.tjcn.org/) |  |
|  | GDP | 1Km | (https://doi.org/10.3974/geodb.2014.01.07.V1) |  |
|  | urbanization rate (UR) |  | Statistical yearbook of Xinjiang (http://www.tjcn.org/) |  |
| climatic | Annual mean temperature (tem) | 0.5° | (https://catalogue.ceda.ac.uk/uuid/c26a65020a5e4b80b20018f148556681) |  |
|  | Annual mean Precipitation (Pre) | 0.5° | (https://catalogue.ceda.ac.uk/uuid/c26a65020a5e4b80b20018f148556681) |  |


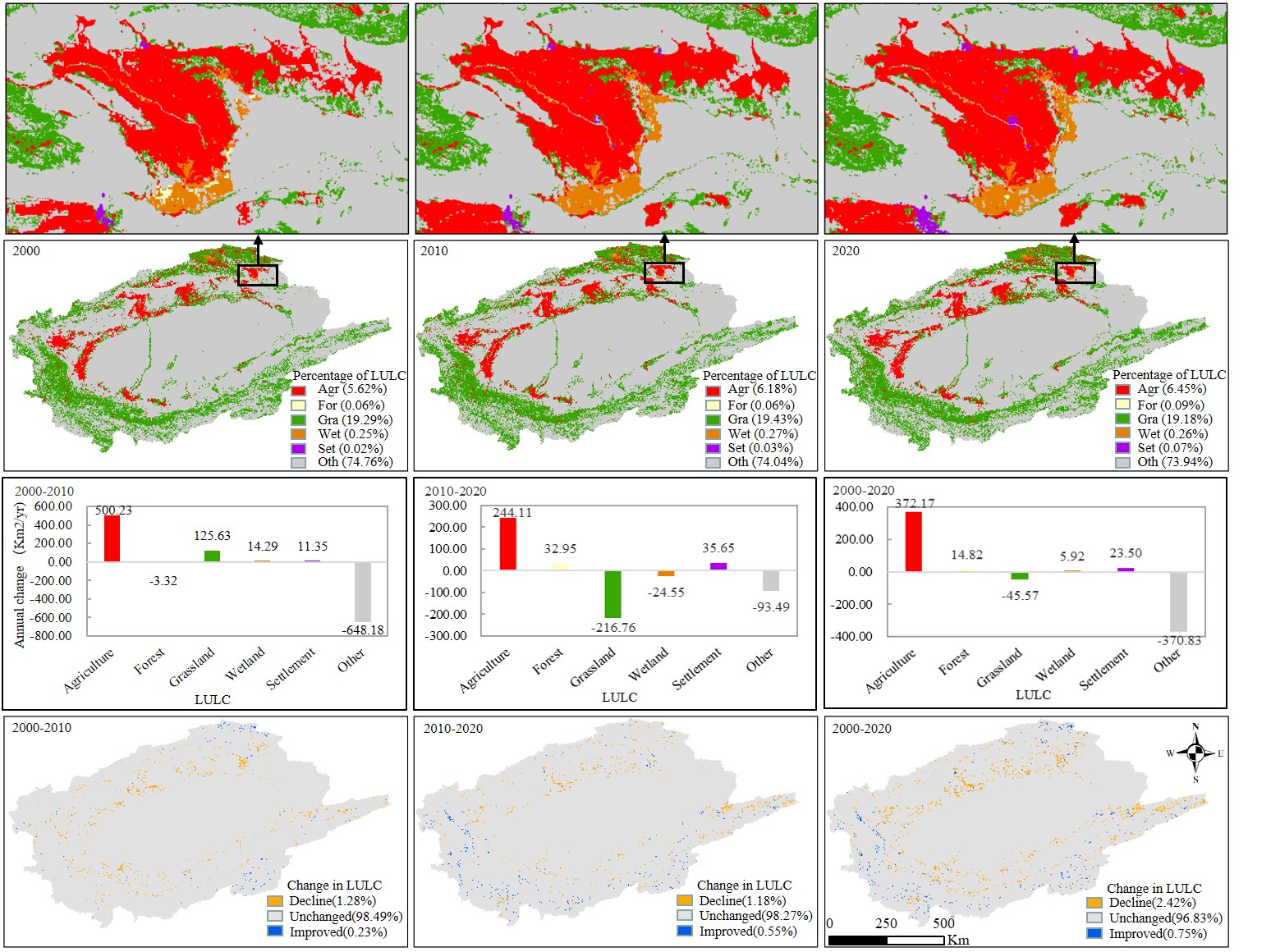


FIGURE 1 Spatial distribution and changes of each LULC class, and the percentage share of each LULC class for 2000-2010, 2010-2020, and 2000-2020 in the TRB. Agr, agricultural land; For, forestland; Gra, grassland; Wet, wetland; Set, settlement. Whereas Oth refers to other types of LULC which include water, bare land, snow and ice.


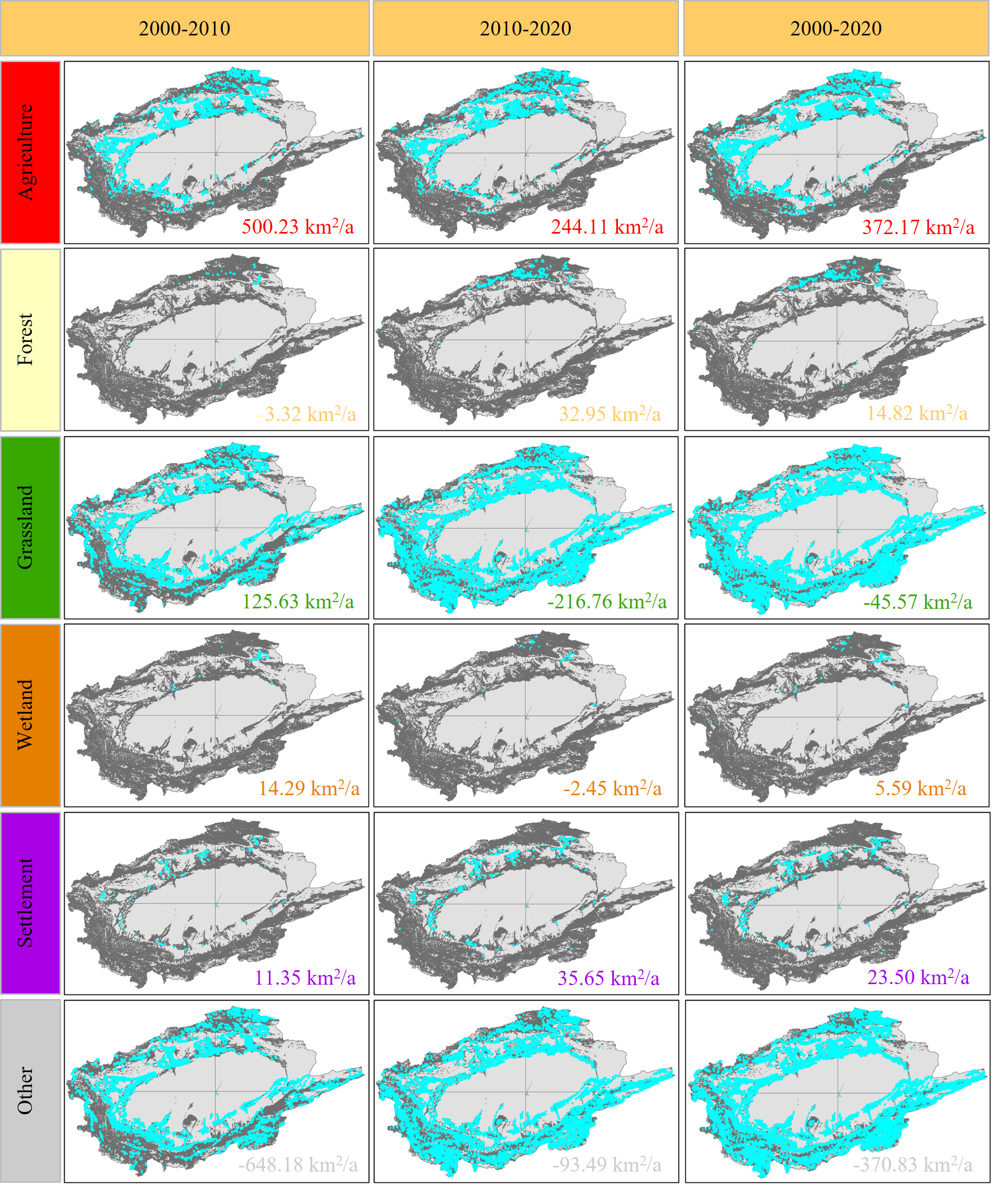


FIGURE 2 Interannual variability and spatial distribution of different LULC types.


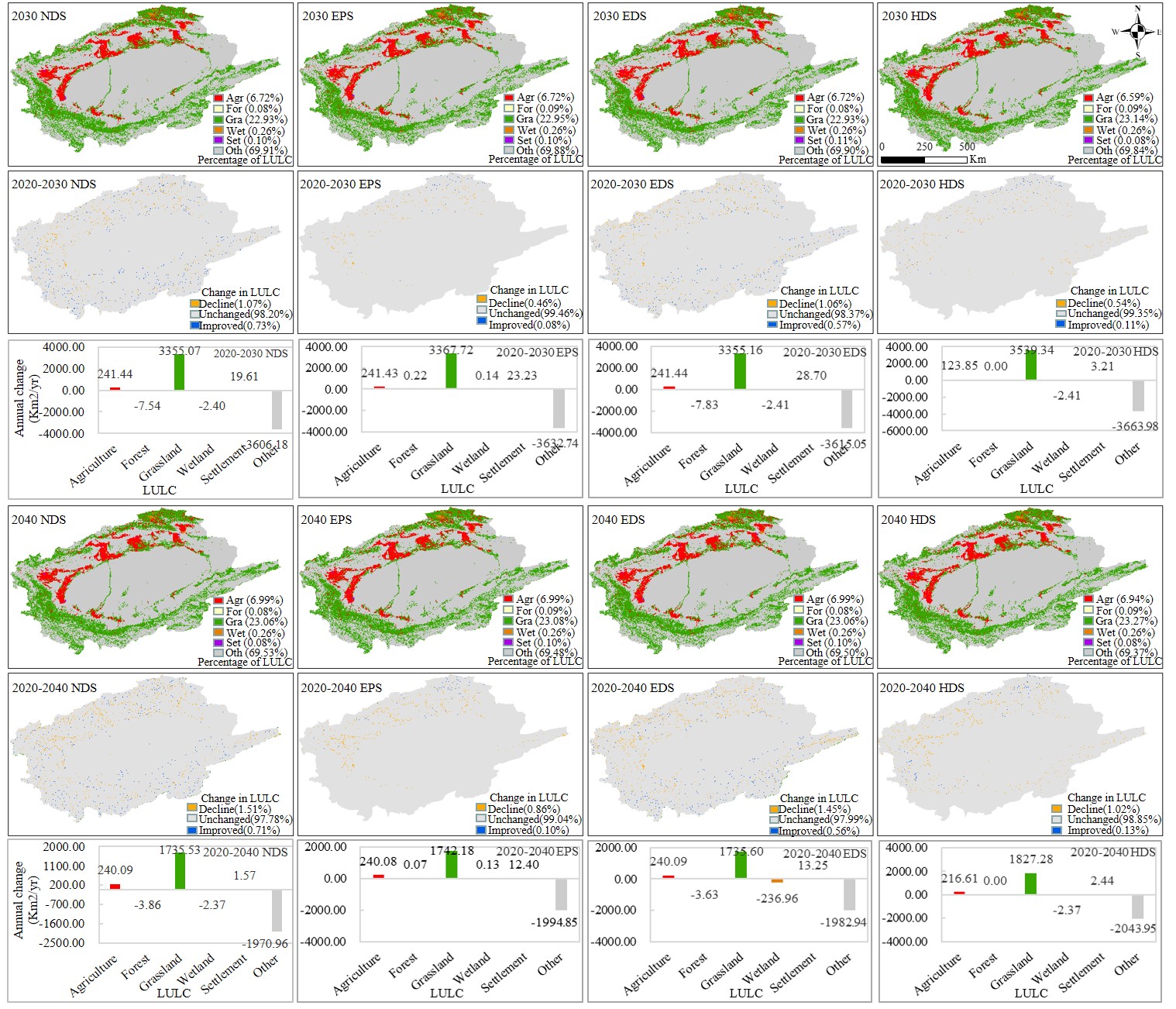


FIGURE 3 Spatial distribution and changes of each projected LULC under different scenarios and their annual change rate per year. NDS, natural development scenario; EDS, economic development scenario; EPS, ecological priority scenario; HDS, harmonious development scenario; Agr, agricultural land; For, forestland; Gra, grassland; Wet, wetland; Set, settlement. Whereas Oth refers to other types of LULC which include water, bare land, snow and ice.

Eq. (A.1):

$$FOM=\frac{B}{A+B+C+D}$$

where A is a region of error because of observed change predicted as persistence, B is a region of accuracy because of observed change predicted as change, C is a region of error because of observed change predicted as changing to an incorrect category, and D is a region of error because of observed persistence predicted as change(Liu et al., 2017).

Eq. (A.2):

$$K\text{a}ppa=\frac{(P_{0}-P_{c})}{(P_{a}-P_{c})}$$

where *P_0_* refers to the probability of correct simulation, *P_c_* refers to the correct proportion of a

prediction simulation, and *P_a_* refers to the probability of an ideal simulation(Guo et al., 2021; Lin et al., 2020).

The probability-of-occurrence is estimated according to the following equation.

Eq. (B.1):

$$sp\left(p,k\right)=\sum_{j} w_{j,k}\times\frac{1}{1+e^{-N_{j}\left(p,q\right)}}$$

Eq. (B.2):

$$N_{j}\left(p,q\right)=\sum w_{i,j}\times x_{i}\left(p,q\right)$$

where *sp*(*p,k*) is the probability-of-occurrence of LULC type *k* on raster cell *p*; *N_j_*(*p,q*) refers to the signal received by neuron *j*; *x_i_*(*p,q*) refers to the *i* variable associated with the input neuron *i* on raster cell *p*; *w_i,j_* is an adaptive weight between the input and hidden layer; and *w_j,k_* is an adaptive weight between the hidden and output layer(Liang et al., 2018; Liu et al., 2017).

The inertia coefficient is defined as:

2

1

−

−

≤

*t*

*k*

*t*

*k*

*D*

*D*

Eq. (B.3):

Eq. (B.4):

Eq. (B.5):

where $Inertia_{k}^{t}$ refers to the inertia coefficient for LULC type *k* at iteration time *t*, and $D_{k}^{t-1}$ refers to the difference between the macro demand and the allocated amount of LULC type *k* until iteration time *t*−1(Liang et al., 2018; Liu et al., 2017).

Neighborhood effects is estimated as follows:

Eq. (B.6):

$\Omega_{p,k}^{t}=\frac{\sum_{N\times N} con\left(c_{p}^{t-1}=k\right)}{N\times N-1}\times w_{k}$ (3)

where $\sum_{N\times N} con\left(c_{p}^{t-1}=k\right)$ refers to the total number of raster cells occupied by the LULC type *k* at the last iteration time *t–*1 within the *N × N* window, and *w_k_* refers to the weight among the different LULC types(Liu et al., 2017).

**References**

Guo, H., Cai, Y., Yang, Z., Zhu, Z., Ouyang, Y., 2021. Dynamic simulation of coastal wetlands for Guangdong-Hong Kong-Macao Greater Bay area based on multi-temporal Landsat images and FLUS model. Ecol. Indic. 125, 107559.

Liang, X., Liu, X., Li, X., Chen, Y., Tian, H., Yao, Y., 2018. Delineating multi-scenario urban growth boundaries with a CA-based FLUS model and morphological method. Landscape Urban Plan. 177, 47-63.

Lin, W., Sun, Y., Nijhuis, S., Wang, Z., 2020. Scenario-based flood risk assessment for urbanizing deltas using future land-use simulation (FLUS): Guangzhou Metropolitan Area as a case study. Sci. Total Environ. 739, 139899.

Liu, X., Liang, X., Li, X., Xu, X., Ou, J., Chen, Y., Li, S., Wang, S., Pei, F., 2017. A future land use simulation model (FLUS) for simulating multiple land use scenarios by coupling human and natural effects. Landscape Urban Plan. 168, 94-116.
